# Prognostic and clinicopathological significance of C-reactive protein in patients with ovarian cancer: a meta-analysis

**DOI:** 10.1186/s12957-023-03290-5

**Published:** 2024-01-03

**Authors:** Wei Zhang, Zongxin Zhang, Lihong Qian

**Affiliations:** 1 Clinical Laboratory, Nanxun District Hospital of Traditional Chinese Medicine, Huzhou, Zhejiang 313009 China; 2 Clinical Laboratory, Huzhou Central Hospital, Affiliated Central Hospital of Huzhou University, The Fifth School of Clinical Medicine Zhejiang Chinese Medical University, Huzhou, Zhejiang 313000 China; 3 Operating Room, Huzhou Central Hospital, Affiliated Central Hospital of Huzhou University, The Fifth School of Clinical Medicine Zhejiang Chinese Medical University, Huzhou, Zhejiang 313000 China

**Keywords:** CRP, Ovarian cancer, Meta-analysis, Clinical management, Prognosis

## Abstract

**Background:**

Many studies have explored the relationship between C-reactive protein (CRP) levels and survival outcomes in patients with ovarian cancer (OC); however, consistent results have not been reported. As such, this meta-analysis was performed to accurately assess the prognostic and clinicopathological roles of CRP in OC.

**Methods:**

The PubMed, Web of Science, Embase, and Cochrane Library databases were systematically searched for relevant studies published from inception to April 7, 2023. The effect of CRP level(s) and OC prognostic outcomes was analyzed by computing the combined hazard ratio (HR) and corresponding 95% confidence interval (CI). Thereafter, the association between CRP level(s) and clinicopathological factors was evaluated using a combined odds ratio (OR) and corresponding 95% CI.

**Results:**

The present meta-analysis included 15 studies comprising 3202 subjects. According to the combined data, higher CRP levels were markedly associated with unfavorable overall survival (OS) (HR 1.23 [95% CI 1.11–1.37]; *p* < 0.001) and progression-free survival (PFS) (HR 1.55 [95% CI 1.30–1.84]; *p* < 0.001) in patients with OC. Furthermore, the results indicated that high CRP levels were significantly correlated with International Federation of Gynecology and Obstetrics (FIGO) stages III–IV (*p* < 0.001), residual tumor size ≥ 1 cm (*p* < 0.001), histological grade 3 (*p* = 0.040), and ascites volume ≥ 500 mL (*p* < 0.001).

**Conclusion:**

The results of this meta-analysis demonstrated that higher serum CRP levels were strongly associated with dismal OS and PFS in subjects with OC. High CRP levels were also significantly associated with clinical factors implicated in tumor aggressiveness and the development of OC.

**Supplementary Information:**

The online version contains supplementary material available at 10.1186/s12957-023-03290-5.

## Background

In recent decades, ovarian cancer (OC), a frequently observed malignancy among females, has been characterized by high mortality and morbidity rates worldwide [1]. OC accounts for 1.6% of newly diagnosed cancer cases and 2.1% of cancer-associated mortality worldwide annually [2]. Approximately 313,959 new cases of OC and 207,252 cases of OC-related death were reported globally in 2020 [3]. Despite the progress in diagnosis, surgery, chemotherapy, radiotherapy, and immunotherapy of OC over the past decade [4, 5], 5-year survival and recurrence rates remain only at 39% and 70%, respectively [6, 7]. Poor prognosis and a high incidence of OC recurrence may, in part, be associated with insufficient efficient markers for prognosis prediction. Consequently, the identification of new and reliable prognostic biomarkers for OC is necessary to inform and support clinical management.

Current evidence has revealed that inflammation and immunity contribute to the initiation, progression, development, and metastasis of cancer [8]. The relationship between chronic inflammation and cancer has attracted increasing attention, and inflammation is now considered to be a facilitating feature [9]. Inflammation can promote tumor progression and metastasis [10]. C-reactive protein (CRP) is an acute-phase protein mostly generated in the liver after infection, inflammation, and tissue injury [11]. As reported by many studies, serum CRP levels are elevated in multiple cancers [12, 13]. Previous investigations have reported that high serum CRP levels predict dismal prognosis in different cancer types, such as breast cancer [14], diffuse large B-cell lymphoma (DLBCL) [15], nasopharyngeal carcinoma [16], renal cell carcinoma [17], and colorectal cancer [18]. Furthermore, current evidence indicates that high CRP levels are associated with an increased risk for OC [19]. According to a multicenter study, CRP is implicated in ovarian carcinogenesis and inflammation and is particularly linked to endometrioid and mucinous carcinomas [19]. Moreover, a previous study suggested that high CRP levels were correlated with OC stage and tumor size [20]. The utility of CRP levels in predicting the prognosis of OC has been widely explored [21–35]; however, consistent results have not been reported. For example, a higher CRP level has been reported to be markedly associated with poor survival of patients with OC in some studies [26, 32, 35]. However, other researchers failed to identify any relationship between CRP and survival in those with OC [24, 31]. As such, we performed a comprehensive literature search to investigate the utility of CRP in accurately predicting the prognosis of patients with OC. Additionally, the relationship between CRP level(s) and the clinicopathological characteristics of patients with OC was also explored.

## Materials and methods

### Study guideline

The present meta-analysis was performed in accordance with the Preferred Reporting Items for Systematic Reviews and Meta-Analyses (PRISMA) guidelines [36]. The PRISMA checklist is provided as Additional file 1. The protocol of this meta-analysis was registered in INPLASY (ID: INPLASY202380097). The link of this protocol is https://inplasy.com/inplasy-2023-8-0097/.

### Ethics statement

This meta-analysis did not require ethics approval because the data did not contain personal information, which precluded any privacy concerns.

### Literature retrieval

The PubMed, Web of Science, Embase, and Cochrane Library databases were searched for relevant studies, published from inception until April 7, 2023, using the following search strategies and terms: (C-reactive protein or C-reactive protein or CRP) and (ovarian cancer or ovarian neoplasm or ovarian carcinoma or ovarian tumor). The detailed search strategies for each database are shown in Additional file 2. The literature search was restricted to studies published in English. In addition, the reference lists of eligible studies were manually searched to identify other potentially relevant works.

### Inclusion and exclusion criteria

Studies were included based on the following criteria: OC diagnosed by pathology, reporting an association between pretreatment CRP levels and any survival outcome in OC, available hazard ratios (HRs) and 95% confidence intervals (CIs) for prognosis or calculability based on available data, a threshold identified to stratify low and high CRP levels, and published in English. Review articles, meeting abstracts, letters, case reports, comments, studies with no survival data, and animal studies were excluded.

### Data acquisition and quality evaluation

Two researchers (WZ and ZZ) reviewed the potentially eligible studies and collected the data. Disagreements were discussed with a third researcher (LQ) until a consensus was reached. The following information was extracted from each of the included studies: first author, publication year, country, sample size, age, study period, International Federation of Gynecology and Obstetrics (FIGO) stage, study center, treatment, threshold CRP level (mg/L), threshold determination approach, survival endpoint, survival analysis, follow-up, and HRs with corresponding 95% CIs. Overall survival (OS) and progression-free survival (PFS) were the primary and secondary outcomes, respectively. The Newcastle–Ottawa Quality Assessment Scale (NOS) was used to evaluate the methodological quality of the included studies [37]. More specifically, study quality was divided into three categories: participant selection (0–4 points), study comparability (0–2 points), and outcome ascertainment (0–3 points), with a total score of 0–9. Studies with NOS scores ≥ 6 were considered to be of high quality.

### Statistical analysis

Combined HR and 95% CI were determined to evaluate whether CRP could be used to predict the prognosis of patients with OC. Heterogeneity across the included studies was explored using Cochran’s *Q* test and the *I*^2^ statistic. Studies with *I*^2^ > 50% and/or *p* < 0.10 indicated obvious heterogeneity; accordingly, combined HR was calculated using a random-effects model; otherwise, a fixed-effects model was used. Subgroup analyses according to different factors were performed to identify potential sources of heterogeneity. In addition, the relationship between CRP level(s) and clinicopathological factors in patients with OC was assessed using a combined odds ratio (OR) and corresponding 95% CI. Funnel plot symmetry was visually inspected to assess publication bias using Begg’s and Egger’s tests. Statistical analysis was performed using Stata version 12.0 (StataCorp LLC, College Station, TX, USA). Differences with *p* < 0.05 were considered to be statistically significant.

## Results

### Literature selection process

**Fig. 1:**
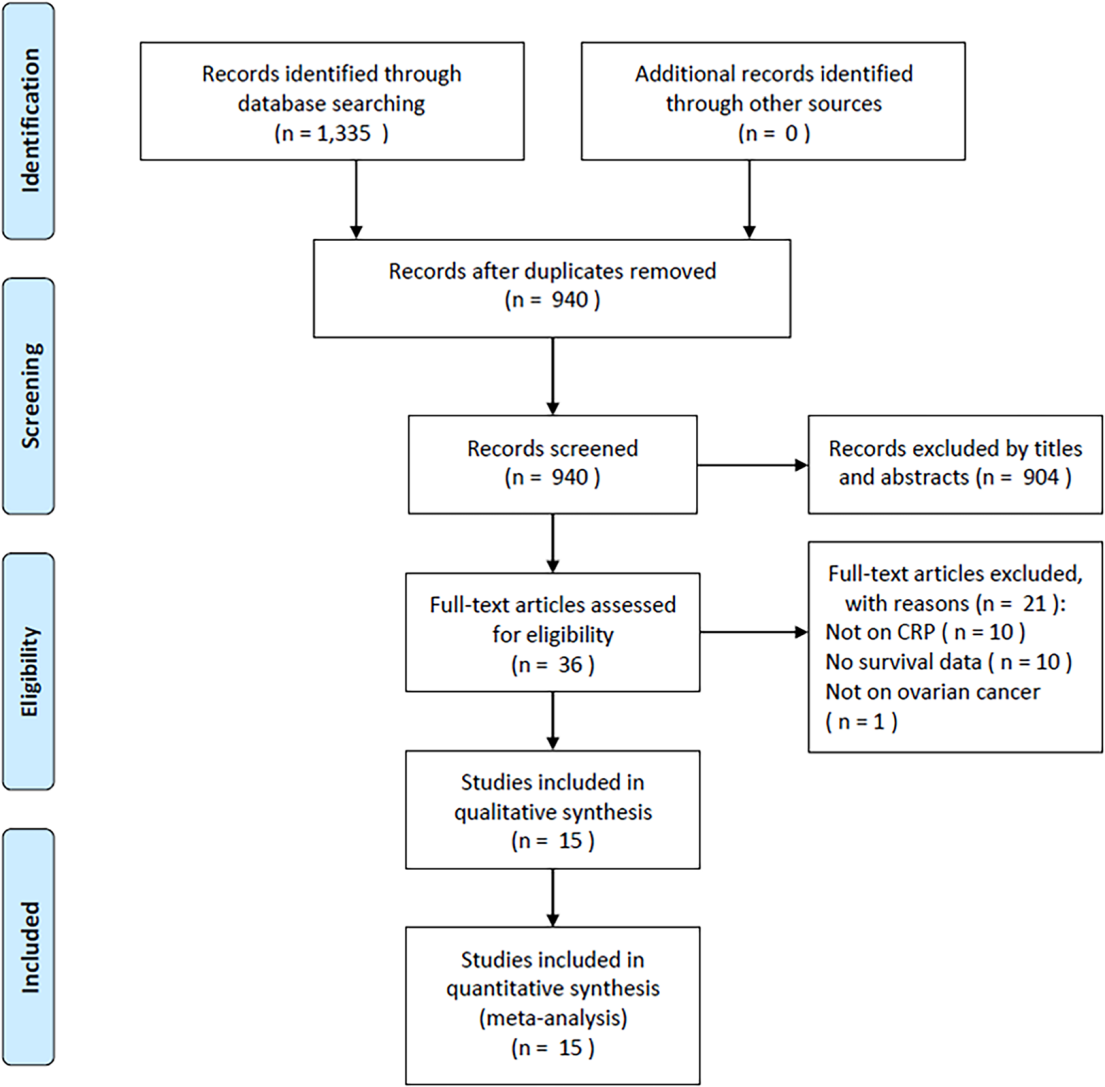
The PRISMA flow diagram of identifying eligible studies

In total, the primary literature search retrieved 1335 articles (Fig. 1), of which 940 were retained after the removal of duplicates. After screening the titles and abstracts, 904 studies were excluded because they were irrelevant or were animal studies, and 36 were further evaluated by full-text examination. Twenty-one studies were excluded because they did not focus on CRP (*n* = 10), did not report survival information (*n* = 10), or did not study patients with OC (*n* = 1). Ultimately, the present meta-analysis included 15 studies comprising 3202 subjects [21–35] (Fig. 1).

### Features of the included studies

The baseline characteristics of the included studies are summarized in Table 1. These studies were published between 1999 and 2023. Six studies were conducted in China [26, 27, 30–32, 35], four in Japan [21, 24, 29, 34], and one each in Austria [22], Australia [23], Poland [25], the USA [28], and Turkey [33]. Each of the included studies was retrospective in design and published in English [21–35]. The sample size ranged from 48–623 (median, 154). Thirteen studies were single-center investigations [21, 23–25, 27–35], and two were multicenter trials [22, 26]. Eleven studies included patients with OC with FIGO stages I–IV [21, 22, 24–27, 30, 32–35], three included those with FIGO stages III–IV [23, 28, 31], and one included OC stage IV [29]. In addition, the threshold CRP level was 3.5–70 mg/L (median, 10 mg/L). Ten studies used receiver operating characteristic (ROC) curve analysis to determine the thresholds [22, 25–27, 29–32, 34, 35], two adopted the 75th percentile value [21, 28], and one each used values reported in the literature [23], mean value [24], and median value [33]. Fourteen articles reported the significance of CRP level in predicting OS in OC [21–33, 35], while seven reported the relationship between CRP and PFS [24, 25, 27, 31, 32, 34, 35]. Eight studies calculated HRs and 95% CIs based on multivariate regression [21, 22, 25, 29, 30, 32, 34, 35], and seven calculated these data using univariate regression [23, 24, 26–28, 31, 33]. The NOS scores of the included studies ranged from 7 to 9 points (median, 8 points), indicating high quality (Table 1).

**Table 1:** Baseline characteristics of included studies

Study	Year	Country	Sample size	Age (years), median (range)	Study duration	FIGO stage	Study center	Treatment	Cutoff value (mg/L)	Cutoff determination	Survival endpoint	Follow-up (months), median (range)	Survival analysis	NOS score
Kodama, J	1999	Japan	120	52 (20–85)	1985–1992	I–IV	Single center	Surgery + chemotherapy	50	75th percentile	OS	1–60	Multivariate	8
Hefler, L. A	2008	Austria	623	60.5	NR	I–IV	Multicenter	Surgery + chemotherapy	10	ROC curve	OS	25.5	Multivariate	8
Sharma, R	2008	Australia	154	63.3 (30–93)	2003–2006	III–IV	Single center	Surgery + chemotherapy	10	Literature	OS	21	Univariate	8
Nakamura, K	2012	Japan	51	60.3 (31–83)	2007–2010	I–IV	Single center	Surgery + chemotherapy	16.7	Mean value	OS, PFS	1–40	Univariate	7
Dobrzycka, B	2013	Poland	118	57.6 (19–78)	2003–2007	I–IV	Single center	Surgery + chemotherapy	11.19	ROC curve	OS, PFS	24.6 (0.8–58.2)	Multivariate	7
Lu, Y	2015	China	107	55 (34–79)	2006–2010	I–IV	Multicenter	Surgery + chemotherapy	8	ROC curve	OS	1–60	Univariate	9
Zhang, W. W	2015	China	190	50.6 (24–76)	2000–2012	I–IV	Single center	Surgery + chemotherapy	10	ROC curve	OS, PFS	43 (2–164)	Univariate	8
Kumar, A	2017	USA	48	68.8	2002–2009	III–IV	Single center	Surgery + chemotherapy	70	75th percentile	OS	1–12	Univariate	7
Utsumi, F	2017	Japan	77	58	2003–2012	IV	Single center	Chemotherapy	5	ROC curve	OS	27.8 (1–188)	Multivariate	7
Li, Y	2019	China	186	59.2	2008–2013	I–IV	Single center	Surgery	6.8	ROC curve	OS	45.5 (2–99.1)	Multivariate	8
Yu, W	2019	China	313	64.4	2010–2017	III–IV	Single center	NAC + surgery	7.4	ROC curve	OS, PFS	1–80	Univariate	7
Chen, K	2020	China	328	51	2014–2019	I–IV	Single center	Surgery + chemotherapy	3.5	ROC curve	OS, PFS	52	Multivariate	8
Sahin, H. O	2020	Turkey	97	51 (24–84)	2012–2019	I–IV	Single center	Surgery + chemotherapy	16	Median value	OS	56 (1–84)	Univariate	8
Komura, N	2021	Japan	308	< 50 years: 101 ≥ 51 years: 207	2007–2016	I–IV	Single center	Surgery + chemotherapy	7.6	ROC curve	PFS	1–120	Multivariate	7
Pan, Q	2023	China	482	51.5 (16–79)	2002–2016	I–IV	Single center	Surgery + chemotherapy	5.15	ROC curve	OS, PFS	49 (3–190)	Multivariate	7

*NR* not reported, *FIGO* International Federation of Gynecology and Obstetrics, *ROC* receiver operating characteristics, *OS* overall survival, *PFS* progression-free survival, *NAC* neoadjuvant chemotherapy, *NOS* Newcastle–Ottawa Scale

### CRP level and OS among patients with OC

**Fig. 2:**
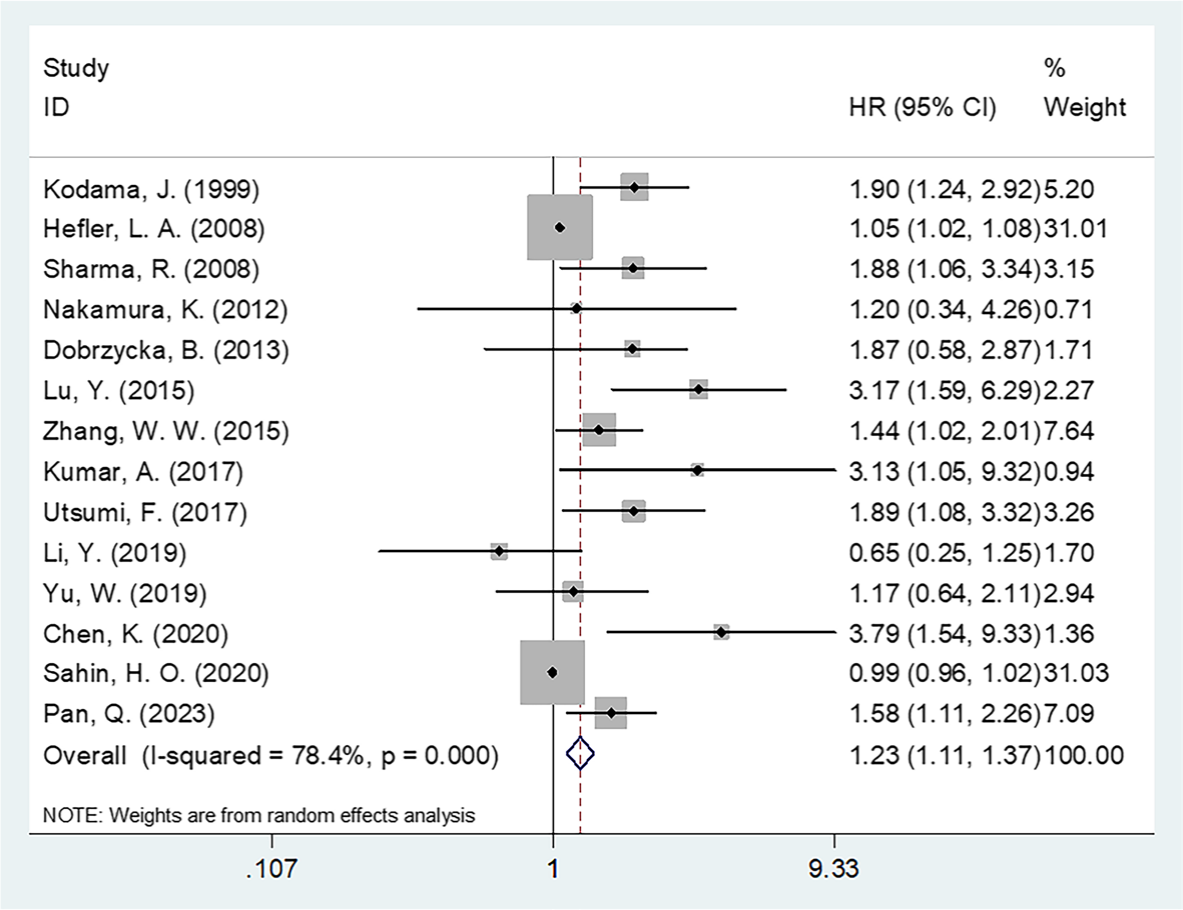
Forest plot of the prognostic role of CRP for OS in patients with OC

In total, 14 studies comprising 2894 subjects [21–33, 35] investigated the utility of CRP levels in estimating OS. A random-effects model was used due to obvious heterogeneity (*I*^2^ = 78.4%, *p* < 0.001). Higher CRP levels demonstrated remarkable utility in predicting poor OS among patients with OC (HR 1.23 [95% CI 1.11–1.37]; *p* < 0.001) (Fig. 2, Table 2). A subgroup analysis was then performed using various factors, including geographical region, sample size, FIGO stage, study center, treatment, threshold CRP, threshold determination method, and survival analysis types. As shown in Table 2, higher CRP levels were still a significant prognostic indicator of poor OS, irrespective of FIGO stage, cutoff value, or survival analysis type (*p* < 0.05). Furthermore, higher CRP levels exhibited a close association with shorter OS in the following subgroups: studies conducted in Asia (HR 1.52 [95% CI 1.13–2.05]; *p* = 0.005); sample size < 150 (HR 1.95 [95% CI 1.24–3.06]; *p* = 0.004); single-center studies (HR 1.53 [95% CI 1.17–1.99]; *p* = 0.002); and treatment using surgery + chemotherapy (HR 1.23 [95% CI 1.10–1.37]; *p* < 0.001) together with threshold determination using ROC curve analysis (HR 1.52 [95% CI 1.14–2.03]; *p* = 0.004) (Table 2).

**Table 2:** Subgroup analysis of the prognostic value of CRP for OS in patients with ovarian cancer

Subgroups	No. of studies	No. of patients	Effects model	HR (95%CI)	*p*	Heterogeneity	
						*I*^2^ (%)	Ph
Total	14	2894	Random	1.23 (1.11–1.37)	< 0.001	78.4	< 0.001
Geographical region							
Asia	10	1951	Random	1.52 (1.13–2.05)	0.005	80.0	< 0.001
Non-Asia	4	943	Random	1.60 (0.97–2.63)	0.067	69.3	0.020
Sample size							
< 150	7	618	Random	1.95 (1.24–3.06)	0.004	82.3	< 0.001
≥ 150	7	2276	Random	1.26 (0.99–1.61)	0.060	63.4	0.018
FIGO stage							
I–IV	10	2302	Random	1.17 (1.05–1.30)	0.004	81.0	< 0.001
III–IV/IV	4	592	Fixed	1.72 (1.25–2.36)	0.001	0	0.397
Study center							
Single center	12	2164	Random	1.53 (1.17–1.99)	0.002	75.8	< 0.001
Multicenter	2	730	Random	1.73 (0.59–5.06)	0.321	89.9	0.002
Treatment							
Surgery + chemotherapy	11	2318	Random	1.23 (1.10–1.37)	< 0.001	81.6	< 0.001
NAC + surgery/chemotherapy/surgery	3	576	Random	1.19 (0.67–2.11)	0.546	57.3	0.096
CRP cutoff value (mg/L)							
< 10	6	1493	Random	1.70 (1.13–2.56)	0.011	63.0	0.019
≥ 10	8	1401	Random	1.10 (1.01–1.20)	0.037	76.8	< 0.001
Cutoff determination							
ROC curve	9	2424	Random	1.52 (1.14–2.03)	0.004	76.0	< 0.001
Median/mean value	2	148	Fixed	0.99 (0.96–1.02)	0.581	0	0.766
75th percentile	2	168	Fixed	2.03 (1.36–3.04)	0.001	0	0.403
Literature	1	154	–	1.88 (1.06–3.34)	0.031	–	–
Survival analysis							
Univariate	7	960	Random	1.54 (1.08–2.20)	0.018	75.7	< 0.001
Multivariate	7	1934	Random	1.53 (1.09–2.15)	0.015	78.1	< 0.001

### CRP level and PFS in patients with OC

**Fig. 3:**
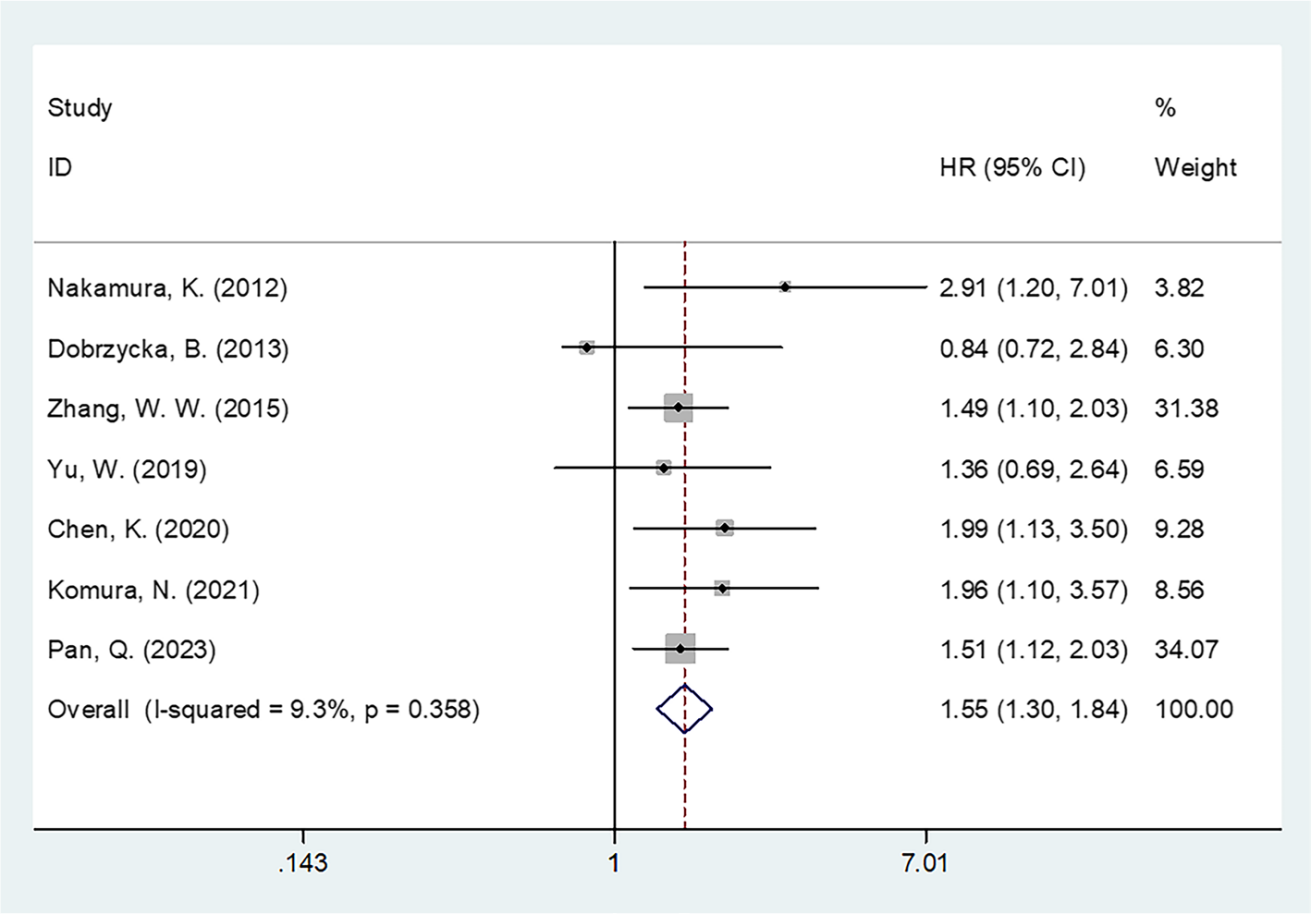
Forest plot of the prognostic role of CRP for PFS in patients with OC

Seven studies enrolling 1790 patients [24, 25, 27, 31, 32, 34, 35] analyzed the effect of CRP level on the prognosis of OC. Owing to non-obvious heterogeneity, a fixed-effects model was adopted (*I*^2^ = 9.3%, *p* = 0.358). Combined data demonstrated that high CRP levels exhibited an obvious relationship with poor PFS in those with OC (HR 1.55 [95% CI 1.30–1.84]; *p* < 0.001) (Table 3, Fig. 3). As revealed by subgroup analysis, the role of CRP in predicting PFS was not influenced by the threshold determination approach or type of survival analysis (*p* < 0.05) (Table 3). Additionally, elevated CRP levels remained the obvious factor predicting dismal PFS for the following subgroups: Asian region (HR 1.61 [95% CI 1.35–1.93]; *p* < 0.001); sample size ≥ 150 (HR 1.53 [95% CI 1.26–1.86]; *p* < 0.001); FIGO stages I–IV (HR 1.56 [95% CI 1.31–1.87]; *p* < 0.001); surgery + chemotherapy treatment (HR 1.56 [95% CI 1.31–1.87]; *p* < 0.001); and threshold CRP < 10 mg/L (HR 1.62 [95% CI 1.29–2.03]; *p* < 0.001) (Table 3).

**Table 3:** Subgroup analysis of the prognostic value of CRP for PFS in patients with ovarian cancer

Subgroups	No. of studies	No. of patients	Effects model	HR (95%CI)	*p*	Heterogeneity	
						*I*^2^ (%)	Ph
Total	7	1790	Fixed	1.55 (1.30–1.84)	< 0.001	9.3	0.358
Geographical region							
Asia	6	1672	Fixed	1.61 (1.35–1.93)	< 0.001	0	0.644
Non-Asia	1	118	–	0.84 (0.42–1.67)	0.618	–	–
Sample size							
< 150	2	169	Random	1.65 (0.83–3.27)	0.152	65.0	0.057
≥ 150	5	1621	Fixed	1.53 (1.26–1.86)	< 0.001	0	0.841
FIGO stage							
I–IV	6	1477	Fixed	1.56 (1.31–1.87)	< 0.001	22.7	0.264
III–IV/IV	1	313	–	1.36 (0.70–2.66)	0.369	–	–
Treatment							
Surgery + chemotherapy	6	1477	Fixed	1.56 (1.31–1.87)	< 0.001	22.7	0.264
NAC + surgery/chemotherapy/surgery	1	313	–	1.36 (0.70–2.66)	0.369	–	–
CRP cutoff value (mg/L)							
< 10	4	1431	Fixed	1.62 (1.29–2.03)	< 0.001	0	0.708
≥ 10	3	359	Random	1.46 (0.85–2.51)	0.167	58.8	0.088
Cutoff determination							
ROC curve	6	1739	Fixed	1.51 (1.27–1.80)	< 0.001	0	0.470
Median/mean value	1	51	–	2.91 (1.20–7.01)	0.018	–	–
Survival analysis							
Univariate	3	554	Fixed	1.56 (1.20–2.04)	0.001	7.5	0.339
Multivariate	4	1236	Fixed	1.54 (1.23–1.93)	< 0.001	32.6	0.217

### Relationship between CRP level and clinicopathological characteristics of patients with OC

**Fig. 4:**
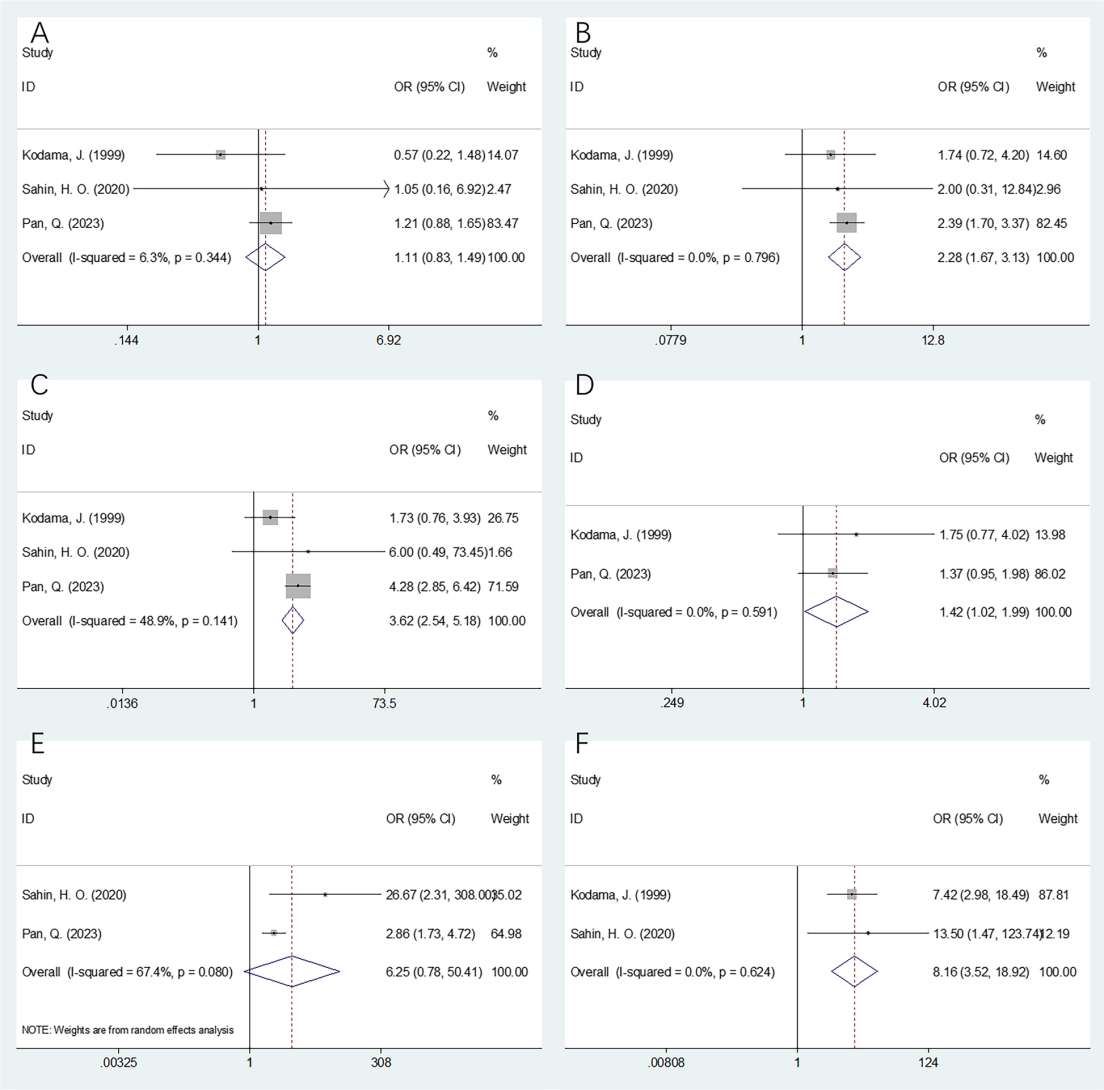
The association between CRP and clinicopathological factors in patients with OC. **A** Age (years) (≥ 51 vs < 50). **B** FIGO stage (III–IV vs I–II). **C** Residual tumor size (cm) (≥ 1 vs < 1). **D** Histologic grade (3 vs 1–2). **E** Preoperative CA125 level (U/mL) (≥ 35 vs < 35). **F** Volume of ascites (mL) (≥ 500 vs < 500)

Three studies including 699 patients [21, 33, 35] explored the relationship between CRP and clinicopathological characteristics such as age (≥ 51 versus vs < 50 years), FIGO stage (III–IV vs I–II), residual tumor size (cm) (≥ 1 vs < 1), histological grade (3 vs 1–2), preoperative carbohydrate antigen (CA) 125 level (≥ 35 vs < 35 U/mL), and volume of ascites (≥ 500 vs < 500 mL). According to the pooled findings reported in Fig. 4 and Table 4, higher CRP levels were remarkably correlated with FIGO stages III–IV (OR 2.28 [95% CI 1.67–3.13]; *p* < 0.001), residual tumor size ≥ 1 cm (OR 3.62 [95% CI 2.54–5.18]; *p* < 0.001), histological grade 3 (OR 1.42 [95% CI 1.02–1.99]; *p* = 0.040), and ascites volume ≥ 500 mL (OR 8.16 [95% CI 3.52–18.92]; *p* < 0.001). However, CRP level did not demonstrate any relationship with age (OR 1.11 [95% CI 0.83–1.49]; *p* = 0.466) or preoperative CA125 level (OR 6.25 [95% CI 0.78–50.41]; *p* = 0.085) (Table 4, Fig. 4).

**Table 4:** The association between CRP and clinicopathological features in patients with ovarian cancer

Variables	No. of studies	No. of patients	Effects model	OR (95%CI)	*p*	Heterogeneity	
						*I*^2^ (%)	Ph
Age (years) (≥ 51 vs < 50)	3	699	Fixed	1.11 (0.83–1.49)	0.466	6.3	0.344
FIGO stage (III–IV vs I–II)	3	699	Fixed	2.28 (1.67–3.13)	< 0.001	0	0.796
Residual tumor size (cm) (≥ 1 vs < 1)	3	699	Fixed	3.62 (2.54–5.18)	< 0.001	48.9	0.141
Histologic grade (3 vs 1–2)	2	602	Fixed	1.42 (1.02–1.99)	0.040	0	0.591
Preoperative CA125 level (U/mL) (≥ 35 vs < 35)	2	589	Random	6.25 (0.78–50.41)	0.085	67.4	0.080
Volume of ascites (mL) (≥ 500 vs < 500)	2	217	Fixed	8.16(3.52–18.92)	< 0.001	0	0.624

### Publication bias

**Fig. 5:**
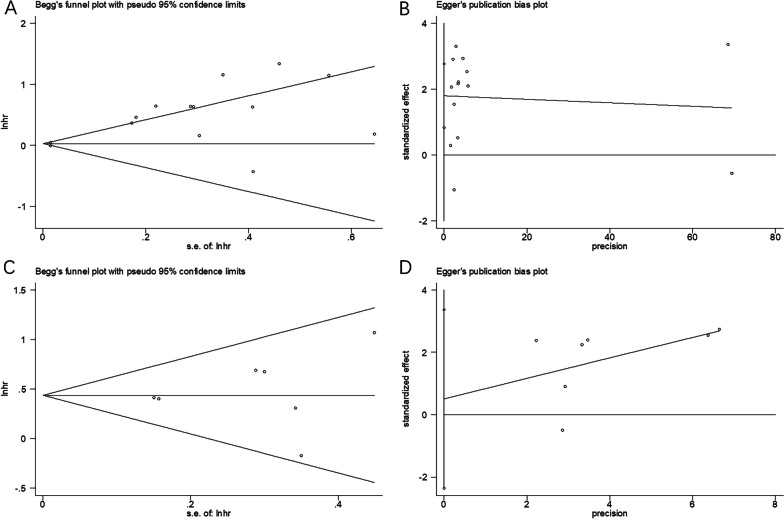
Publication bias for OS and PFS. **A** Begg’s test for OS, *p* = 0.913. **B** Egger’s test for OS, *p* = 0.761. **C** Begg’s test for PFS, *p* = 0.881. **D** Egger’s test for PFS, *p* = 0.666

Funnel plots, together with Begg’s and Egger’s tests, were used to investigate publication bias. Visual inspection of the funnel plots revealed no significant asymmetry in OS or PFS (Fig. 5). Moreover, the findings also demonstrated no obvious publication bias with regard to OS (*p* = 0.913 and *p* = 0.761 according to Begg’s and Egger’s tests, respectively) and PFS (*p* = 0.881 and *p* = 0.666 according to Begg’s and Egger’s tests, respectively). Based on these findings, publication bias was not detected in the included studies.

## Discussion

CRP, a frequently used inflammatory biomarker, is produced in the liver and atherosclerotic plaques. Its role in predicting prognosis in patients with OC has been widely analyzed; however, consistent results have not been reported [21–35]. This study combined data from 15 studies involving 3202 subjects to precisely determine the prognostic utility of CRP levels for predicting prognosis in OC. Our results indicated that elevated CRP levels were markedly associated with shortened OS and inferior PFS in patients with OC. Furthermore, higher CRP levels exhibited a significant relationship with advanced FIGO stage, larger residual tumor size, higher histological grade, and ascites volume ≥ 500 mL. Collectively, these data suggest that elevated CRP level is a prognostic marker for poor short- and long-term survival in patients with OC. Increased CRP levels are also predictive of clinicopathological factors, indicating high disease aggressiveness. To our knowledge, this is the first meta-analysis to investigate whether CRP levels can be used to predict the prognosis of patients with OC.

Higher CRP levels are associated with tissue damage, infection, atherosclerosis, arterial hypertension, obesity, diabetes, and/or cancers [38]. The mechanisms underlying the relationship between high CRP levels and poor OC survival are discussed below. First, chronic and persistent inflammation may lead to carcinogenesis or angiogenesis, which promotes tumor cell proliferation [39]. In particular, certain inflammatory cells can generate cytokines and chemokines in the blood, such as interleukin (IL)-6, IL-8, and tumor necrosis factor-α, which promote the production of CRP in the liver [40]. Second, inflammation can promote tumor development by generating growth factors to sustain cell growth and survival, limit cell death, and produce proangiogenic factors that accelerate neovascularization [41]. Importantly, inflammation in the tumor microenvironment may be reflected by circulating CRP levels and proteins related to early inflammation and have important effects [42]. Third, as supported by increasing evidence, inflammatory factors, such as CRP, are produced by hepatocytes after trauma, infection, and cancer; moreover, they can also be produced by cancer cells [43, 44]. Therefore, CRP level is an easy and credible marker for predicting the prognosis of patients with OC.

In the current meta-analysis, we included 15 studies and expected CRP to be a significant prognostic marker in patients with OC for the following reasons. Previous evidence suggests a biological function of CRP in ovarian carcinogenesis [19, 20]. Second, the included studies provided controversial results regarding the prognostic role of CRP in OC [21–35]. More than one-half of the studies yielded positive results. Third, the significant correlation among FIGO stage, tumor size, and histological grade also met our expectations because these results were in accordance with those of a previous study [20].

Recently, many meta-analyses have explored whether CRP can be used to predict the prognosis of different solid tumors [45–47]. According to a meta-analysis including 16 studies by Zhou et al. [45], higher CRP levels were associated with worse OS, cancer-specific survival, and PFS in prostate cancer. In a meta-analysis of 1287 subjects, Chen et al. [46] reported that patients with metastatic colorectal cancer with higher CRP levels exhibited markedly reduced OS relative to those with lower CRP levels. Based on a meta-analysis including 4449 subjects, Chen et al. [48] reported that higher CRP levels predicted dismal OS, cancer-specific survival, and PFS for head and neck squamous cell carcinoma. A recent meta-analysis of 3000 subjects indicated that higher CRP levels before treatment were associated with poor OS and PFS in diffuse large B-cell lymphoma [49]. Another meta-analysis of 5215 patients revealed that elevated serum CRP levels were associated with worse OS and distant metastasis-free survival in nasopharyngeal carcinoma [50]. Our findings in OC confirmed the prognostic value of CRP for additional cancers.

The present investigation had some limitations. First, the included studies had a retrospective design, and some HRs were calculated based on univariate regression, possibly causing an overestimation of effect sizes. Second, there was inherent heterogeneity in OS―likely due to the retrospective design of the included studies―which persisted after applying the random-effects model. Third, the threshold CRP level and threshold determination approaches were not uniform among the included studies. Therefore, large prospective studies using a standard threshold CRP level should be conducted for further validation.

The current meta-analysis is the first to identify the prognostic and clinicopathological roles of CRP in OC by integrating data from 15 studies. Future studies should focus on the optimal CRP cutoff value for patients with OC. Furthermore, clinical assessment tools that incorporate CRP levels should be developed to predict survival outcomes in patients with OC.

## Conclusions

In conclusion, the results of the present study demonstrated that elevated serum CRP levels predicted poor OS and inferior PFS in patients with OC. High CRP levels were also significantly associated with clinical factors implicated in tumor aggressiveness and development. Therefore, CRP level could be adopted as an easy and credible marker to predict prognosis in patients with OC.

### Supplementary Information

**Additional file 1.** The PRISMA checklist of this meta-analysis.

** Additional file 2.** The detailed literature search strategies for each database.

## Data Availability

The data that support the findings of this study are available from the corresponding author upon reasonable request.

## Abbreviations

CRP: C-reactive protein
OC: Ovarian cancer
HR: Hazard ratio
CI: Confidence interval
OR: Odds ratio
OS: Overall survival
PFS: Progression-free survival
FIGO: International Federation of Gynecology and Obstetrics
NOS: Newcastle-Ottawa Quality Assessment Scale
DLBCL: Diffuse large B-cell lymphoma
