# Supplementary material for: Prognostic and clinicopathological significance of C-reactive protein in patients with ovarian cancer: a meta-analysis
Source: World J Surg Oncol. 2024 Jan 3;22:8. doi: 10.1186/s12957-023-03290-5 (PMC10763048; doi:10.1186/s12957-023-03290-5)
Supplement: Supplementary file 2 — Additional file 2. The detailed literature search strategies for each database. [file 12957_2023_3290_MOESM2_ESM.docx]

**Supplementary file 2. The detailed search strategies for each database.**

**1. Search strategies for PubMed:**

Search: (C-reactive protein or C-reactive protein or CRP) and (ovarian cancer or ovarian neoplasm or ovarian carcinoma or ovarian tumor) Sort by: Most Recent

("c reactive protein"[MeSH Terms] OR ("c reactive"[All Fields] AND "protein"[All Fields]) OR "c reactive protein"[All Fields] OR "c reactive protein"[All Fields] OR ("c reactive protein"[MeSH Terms] OR ("c reactive"[All Fields] AND "protein"[All Fields]) OR "c reactive protein"[All Fields] OR "c reactive protein"[All Fields]) OR ("curr res psychol"[Journal] OR "crp"[All Fields])) AND ("ovarian neoplasms"[MeSH Terms] OR ("ovarian"[All Fields] AND "neoplasms"[All Fields]) OR "ovarian neoplasms"[All Fields] OR ("ovarian"[All Fields] AND "cancer"[All Fields]) OR "ovarian cancer"[All Fields] OR ("ovarian neoplasms"[MeSH Terms] OR ("ovarian"[All Fields] AND "neoplasms"[All Fields]) OR "ovarian neoplasms"[All Fields] OR ("ovarian"[All Fields] AND "neoplasm"[All Fields]) OR "ovarian neoplasm"[All Fields]) OR ("ovarian neoplasms"[MeSH Terms] OR ("ovarian"[All Fields] AND "neoplasms"[All Fields]) OR "ovarian neoplasms"[All Fields] OR ("ovarian"[All Fields] AND "carcinoma"[All Fields]) OR "ovarian carcinoma"[All Fields]) OR ("ovarian neoplasms"[MeSH Terms] OR ("ovarian"[All Fields] AND "neoplasms"[All Fields]) OR "ovarian neoplasms"[All Fields] OR ("ovarian"[All Fields] AND "tumor"[All Fields]) OR "ovarian tumor"[All Fields]))

Translations

C-reactive protein: "c-reactive protein"[MeSH Terms] OR ("c-reactive"[All Fields] AND "protein"[All Fields]) OR "c-reactive protein"[All Fields] OR "c reactive protein"[All Fields]

C-reactive protein: "c-reactive protein"[MeSH Terms] OR ("c-reactive"[All Fields] AND "protein"[All Fields]) OR "c-reactive protein"[All Fields] OR "c reactive protein"[All Fields]

CRP: "Curr Res Psychol"[Journal:__jid101697883] OR "crp"[All Fields]

ovarian cancer: "ovarian neoplasms"[MeSH Terms] OR ("ovarian"[All Fields] AND "neoplasms"[All Fields]) OR "ovarian neoplasms"[All Fields] OR ("ovarian"[All Fields] AND "cancer"[All Fields]) OR "ovarian cancer"[All Fields]

ovarian neoplasm: "ovarian neoplasms"[MeSH Terms] OR ("ovarian"[All Fields] AND "neoplasms"[All Fields]) OR "ovarian neoplasms"[All Fields] OR ("ovarian"[All Fields] AND "neoplasm"[All Fields]) OR "ovarian neoplasm"[All Fields]

ovarian carcinoma: "ovarian neoplasms"[MeSH Terms] OR ("ovarian"[All Fields] AND "neoplasms"[All Fields]) OR "ovarian neoplasms"[All Fields] OR ("ovarian"[All Fields] AND "carcinoma"[All Fields]) OR "ovarian carcinoma"[All Fields]

ovarian tumor: "ovarian neoplasms"[MeSH Terms] OR ("ovarian"[All Fields] AND "neoplasms"[All Fields]) OR "ovarian neoplasms"[All Fields] OR ("ovarian"[All Fields] AND "tumor"[All Fields]) OR "ovarian tumor"[All Fields]

**2. Search strategies for Web of Science:**

(C-reactive protein or C-reactive protein or CRP) and (ovarian cancer or ovarian neoplasm or ovarian carcinoma or ovarian tumor) (all fields)

**3. Search strategies for Embase:**

('c-reactive protein'/exp OR 'c-reactive protein' OR ('c reactive' AND ('protein'/exp OR protein)) OR 'crp'/exp OR crp) AND ('ovarian cancer'/exp OR 'ovarian cancer' OR (ovarian AND ('cancer'/exp OR cancer)) OR 'ovarian neoplasm'/exp OR 'ovarian neoplasm' OR (ovarian AND ('neoplasm'/exp OR neoplasm)) OR 'ovarian carcinoma'/exp OR 'ovarian carcinoma' OR (ovarian AND ('carcinoma'/exp OR carcinoma)) OR 'ovarian tumor'/exp OR 'ovarian tumor' OR (ovarian AND ('tumor'/exp OR tumor)))

**4.** **Search strategies for Cochrane Library:**

(C-reactive protein or C-reactive protein or CRP) and (ovarian cancer or ovarian neoplasm or ovarian carcinoma or ovarian tumor) in Title Abstract Keyword
